# Gaps and Controversies in Feline Food Palatability

**DOI:** 10.3390/ani16172721

**Published:** 2026-09-01

**Authors:** Yolandi van der Vyver, Aman Paul, Christophe Blecker, Sabine Danthine

**Affiliations:** 1 AFB International, 5342 LZ Oss, The Netherlands; apaul@afbinternational.com; 2 TERRA Teaching and Research Centre, Gembloux Agro-Bio Tech, University of Liège, 5030 Gembloux, Belgium

**Keywords:** acceptance, cat, feline, palatability, pet food, preference

## Abstract

Feline food palatability is fundamental to ensure nutritional intake and the commercial success of pet foods, yet the field has several gaps, unresolved contradictions, and methodological inconsistencies. A literature search identified 141 relevant publications. Critical evaluation of this body of work revealed four recurring areas of tension: the inconsistent definition of palatability, where the same term evaluates different constructs; contradictory behavioral indicators, where the same behaviors (e.g., nose-licking) are variably interpreted as positive or negative across studies; methodological fragmentation arising from overarching test selection, study design implementation, and inconsistent or selective reporting; and the disconnect between owners’ perception of palatability and objectively measured feline responses. This critical narrative review discusses the origins of these inconsistencies, their implications for cross-study comparison, and their combined effect on the reliability of existing evidence. Resolving these contradictions may benefit from a proposed integrative framework for defining palatability, a standardized ethogram for behavioral indicators, and a decision framework for selecting assessment methodologies. These approaches could improve the comparability and reliability of future studies, but their effectiveness requires validation.

## Simple Summary

Cats may refuse to eat nutritionally balanced foods if they do not find them palatable, making food palatability important for both cat health and the success of cat food products. However, research on cat food palatability contains several contradictions and inconsistencies that make findings difficult to compare and interpret. This review looks at four key areas of uncertainty. First, the term “palatability” is used to describe different concepts across studies. Second, the same feeding behaviors, such as nose-licking, are often interpreted as either positive or negative indicators of food acceptance. Third, differences in testing methods, study design, and reporting reduce consistency between studies. Finally, owners’ perceptions of how much their cats enjoy food do not always match how much their cat consumes. This review highlights the need for clearer definitions, standardized behavioral measures, and more consistent testing approaches to improve future research on cat food palatability.

## 1. Introduction

The global cat food market is projected to exceed USD 92 billion in 2031, reflecting the growing demand for products that are both nutritionally complete and consistently accepted by cats [1]. Palatability is a primary determinant of voluntary intake and is, therefore, widely recognized as a critical factor in the commercial success of pet food products [2]. For cats, obligate carnivores with specific dietary requirements and strong sensory discrimination, palatability is fundamental. Even nutritionally complete diets may be rejected if they are insufficiently palatable, reducing voluntary intake and contributing to adverse health consequences [3,4]. As a result, the pet food industry allocates substantial resources to palatability testing and product optimization [5].

The study of feline palatability dates back to the mid-20th century [6], with subsequent work in the 1990s establishing the cat’s role as an obligate carnivore with specialized taste receptors [7,8]. While these foundational concepts have guided feline food testing and formulation for decades, and were followed by several comprehensive reviews of sensory drivers [3,9], assessment methods [2,10], and food formulation [11], there remains a lack of synthesis regarding the methodological and conceptual factors that contribute to inconsistent findings across feline palatability research. At the core of these inconsistencies is the absence of a universally accepted definition of palatability. Depending on the study, it is described as the sensory properties of the food, the animal’s voluntary food intake, or the hedonic experience associated with consumption. This lack of conceptual agreement is accompanied by broader inconsistencies throughout the literature. Behavioral indicators regarded as evidence of food acceptance in one study are interpreted as indicators of aversion in another, while reviews of seasonal variation in food intake are often contradictory. These inconsistencies have implications that extend beyond academic research. Differences in how palatability is defined, measured, and interpreted make it difficult to compare studies, identify the sensory and behavioral drivers of feline food preference, and select appropriate methodologies for evaluating palatability. Resolving these issues would improve study comparability, strengthen evidence-based product development, and help distinguish genuine feline preference from owner perception.

Therefore, this review will consider four key areas of unresolved tension in feline palatability research: (i) The inconsistent definition of palatability and the development of an integrative framework. (ii) Contradictions in behavioral indicators and the need for a standardized ethogram. (iii) Methodological fragmentation and a decision framework for assessment methodologies. (iv) What we term the “owner perception paradox”—separating pet palatability from owner purchasing intent. For each area, we identify the specific gap, review examples, and propose concrete directions for future research.

## 2. Materials and Methods

The Scopus database was used as the principal search system to identify inconsistencies within the feline palatability literature [12]. Scopus was selected as the primary bibliographic database as it is one of the largest abstract and citation databases of the peer-reviewed literature, containing over 100 million records from more than 7000 publishers [12,13]. Its broad coverage of animal science, veterinary medicine, agriculture, and food science makes it well-suited for identifying the relevant literature in the multidisciplinary field of feline palatability.

Searches were conducted within article titles, abstracts, and keywords. An initial search of the term “palatability” yielded 12,635 English-language records; however, as the main focus of this review is feline food palatability, the search terms were refined to “palatability” AND (“cat” OR “feline”), resulting in 215 records, of which 200 were published in English. The titles and abstracts of the retrieved records were screened for relevance, and studies were excluded where the term “CAT” referred to an unrelated acronym, palatability was only briefly mentioned, or the research focused solely on the acceptance of medicinal products. Subsequently, 141 publications were retained and critically reviewed to identify recurring themes, inconsistencies, and methodological challenges. Thereafter, similar inconsistencies were grouped through an interpretive synthesis, resulting in four overarching thematic areas that form the structure of this review. Of the 141 publications critically reviewed, 52 were ultimately cited within the review as directly informing or illustrating the themes and issues discussed.

This critical narrative review specifically examined the use and interpretation of the term “palatability” in the feline literature; therefore, the search strategy was centered on this term. Consequently, relevant studies using alternative terminology (e.g., preference, acceptance, or feeding motivation) were potentially overlooked during the primary search. To minimize this limitation, additional publications were identified through targeted searches and citation tracking. In total, 97 publications were cited in this review, comprising 52 publications identified through the primary Scopus search and 45 additional publications identified through targeted searches and citation tracking.

## 3. The Definitional Problem

The term palatability was originally used to describe a person’s behavior and desire to eat, and was subsequently incorporated into experimental nutrition, particularly animal studies. Hill et al. [14] defined palatability as “the sight of highly preferred food markedly increased subjects’ rated desire to eat”. Subsequent work revealed that palatability is more complex and cannot be defined by desire alone. Kissileff [15] proposed to separate “intrinsic palatability” (the sensory properties of the food) from “learned palatability” (prior experiences and post-ingestive consequences). This is an important shift: instead of viewing palatability solely as a property of the food itself, it also considers the learning context and the attributes of the individual consuming it. Around the same time, Rogers [16] further refined the concept, showing the difference between enjoying the taste and enjoying eating. Thus, palatability is a hedonic (pleasure-based) experience, not just sensory. Despite these improvements, Yeomans et al. [17] noted a “lack of consensus on the nature of palatability”. This lack of agreement is more than a linguistic issue. The way palatability is defined directly influences how it is measured and interpreted. If palatability is considered a biological need, food intake and motivation will be emphasized. Conversely, if it is viewed as a hedonic process, then preference- or reward-related behaviors are more relevant. As a result, studies may use the same term while evaluating fundamentally different constructs, making comparisons across disciplines and species difficult [17].

**Figure 1:**
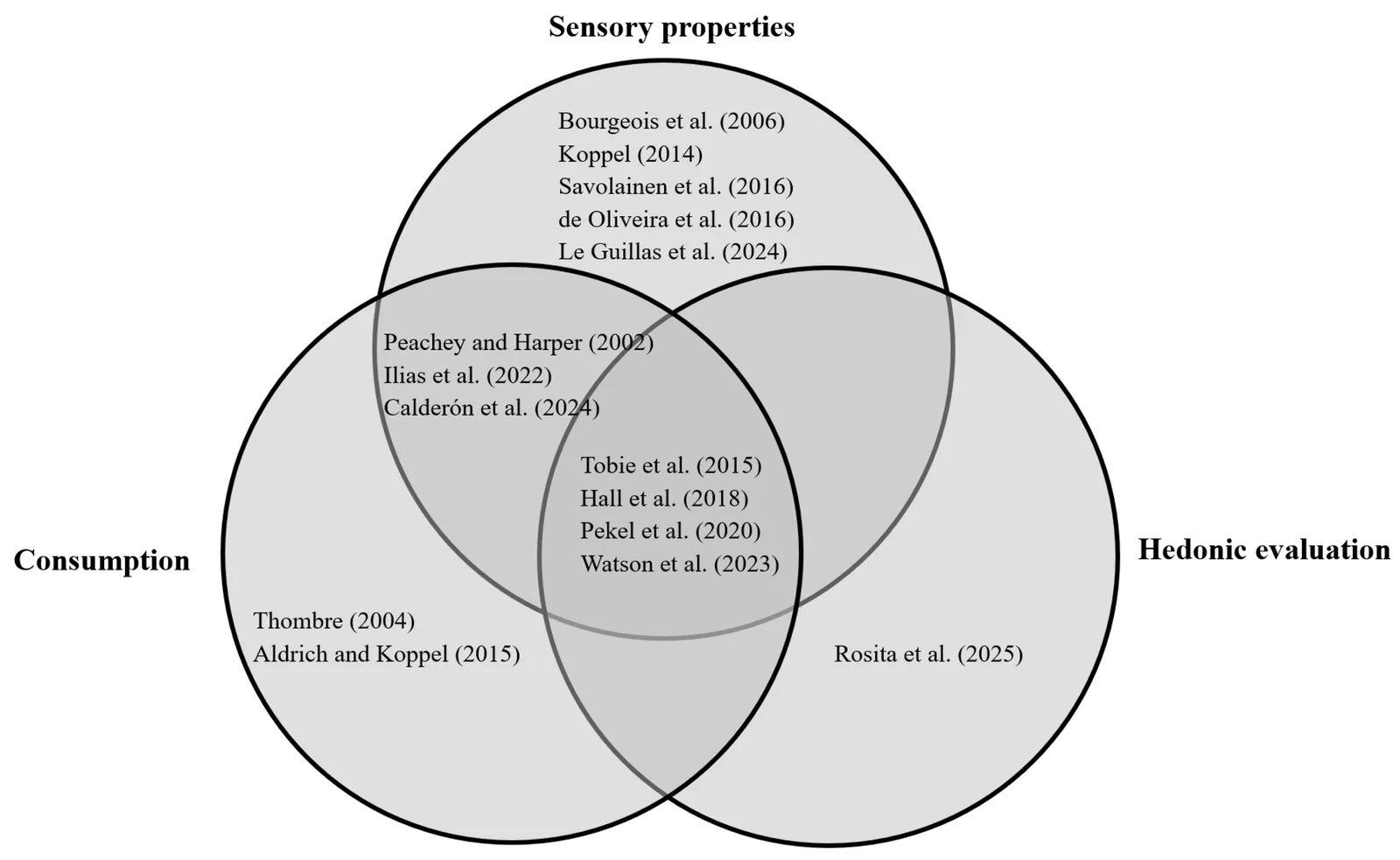
Conceptual definitions of palatability in pet nutrition: a literature synthesis of sensory, consumption, and hedonic perspectives [2,3,9,10,11,18,28,35,36,37,38,39,40,41,42].

The same lack of consensus is evident in pet nutrition [18]. Historical definitions of pet food palatability focused on observed behavior, such as intake or acceptance [2,18,19]. However, this poses a fundamental problem; although highly palatable foods are often consumed in greater quantities, intake is also influenced by factors independent of palatability, including hunger and satiety [20,21,22], health status [23,24,25], neuter status [26], feeding environment [27,28,29], food availability [30,31,32], and prior dietary experiences [33,34]. This definition has since been extended by emphasizing that acceptance is also dependent on the animal’s sensory perception of the food [35,36,37]. Yet, some authors [11,28,38,39,40] solely focus on the sensory properties of the food to define palatability. Until 2015, the definition of pet food palatability was predominantly focused on sensory and consumption attributes. The more recent literature indicates a shift to integrated hedonic concepts, such as defining palatability in terms of pleasure perception, liking, and attractiveness of the food [3,9,10,41]. Watson et al. [9] specifically indicate that palatability is linked to pleasure instead of physiological need, echoing earlier research in human palatability. This is further supported by Rosita et al. [42], who define palatability only as “the degree to which an animal likes food”. It is also worth clarifying what palatability is not: it is not a single sensory attribute, nor a simplistic measure determined by flavor alone [18]. Consequently, there remains uncertainty regarding whether palatability should be regarded as a sensory property of the food, a behavioral outcome, a hedonic response, or a combination of these factors, as seen in Figure 1.

**Figure 2:**
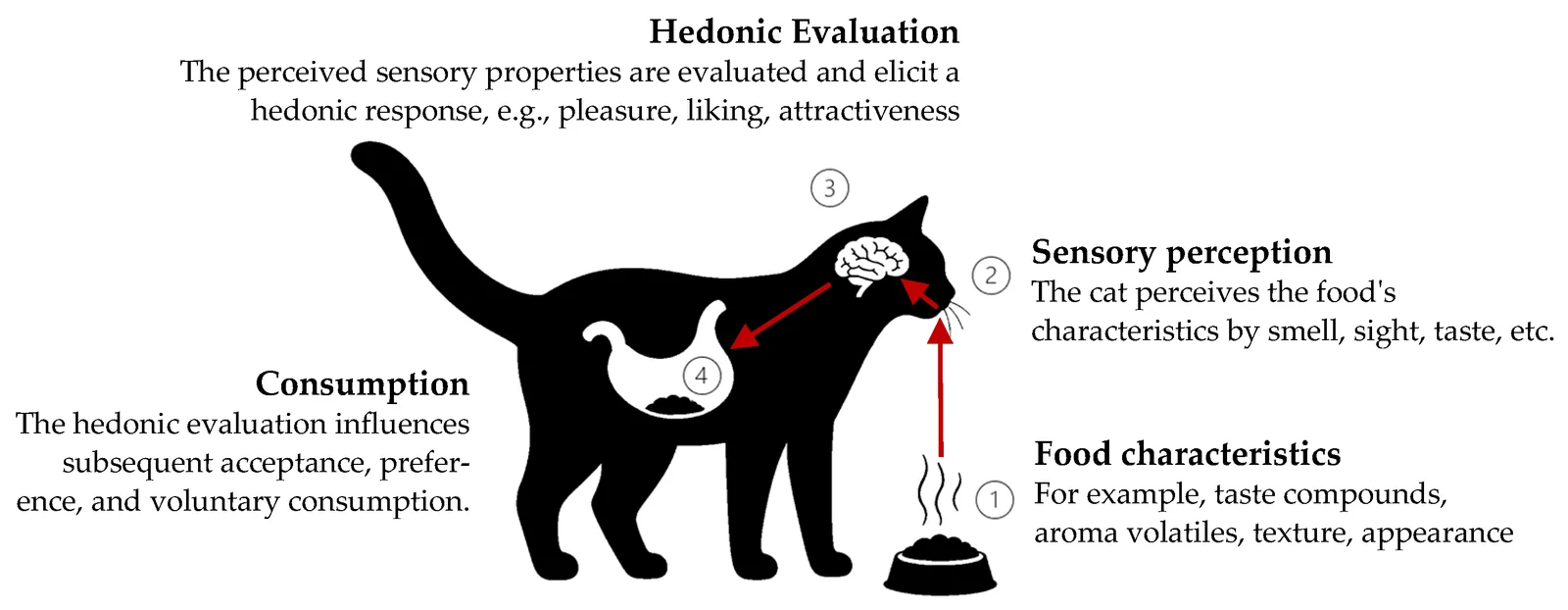
Integrative directional model of feline food palatability.

Rather than treating these perspectives as competing definitions, they may be viewed as complementary components of a broader construct. Within this framework, the sensory dimension refers to the perception of food characteristics such as taste, aroma, texture, and appearance; the hedonic dimension refers to the pleasure, liking, or attractiveness elicited by these sensory perceptions; and the consumption dimension refers to observable voluntary acceptance, preference, and intake behaviors through which palatability is inferred. Importantly, consumption should not be regarded as synonymous with palatability, because it is also influenced by physiological [20,21,22,23,24,25,26], environmental [27,28,29], and experiential factors [33,34,38] independent of the hedonic value of the food. Accordingly, palatability can be conceptualized as an integrative process in which the sensory properties of a food are perceived and hedonically evaluated by the animal, influencing subsequent acceptance, preference, and consumption. This directional relationship is illustrated in Figure 2.

## 4. Contradicting Behavioral Indicators

Investigating feline behavioral indicators to assess responses has been used since 1979 [43] and, more recently, to obtain a complete picture of palatability alongside consumption data [9,10]. The literature has identified a set of behavioral indicators (before, during, and after consumption) that can be broadly categorized as positive or negative with respect to the palatability of the food provided. Positive indicators include an increase in the duration of having the eyes less than 50% open [4], increased lip licking [4,21,39], more frequent mouth smacks [4], and more frequent tongue protrusions [4]. Negative indicators include flicking of the ears backwards or flicking of the tail [39], more frequent tongue-protrusion gapes [4], and a non-significant trend towards the combined behavior of pawing the floor, shaking the head, and limb flailing [44]. Some behavioral indicators, such as posture (sitting or standing) [21,22], twitching of the back skin [39], and licking of the paws [21,44] do not seem to correlate with the palatability of the diet, while playing with the food may indicate satiety rather than palatability [45]. This conventional categorization has facilitated schematic overviews of the relationships between food intake and behavioral responses in cats, such as those presented by Pekel et al. [3] (Figure 5). While these summaries provide a valuable overview, they inevitably simplify a more complex body of literature. A closer examination reveals that several behavioral indicators cannot be consistently classified as purely positive or negative, and that several controversies and details are overlooked in such displays. For example, face grooming is seen as a positive indicator after consuming palatable food [21], whereas body grooming—often noted after consuming less palatable foods or after a period of stress—is seen as a negative indicator [39]. Consequently, classifying “grooming” as a single behavioral indicator may obscure these important distinctions and lead to oversimplified interpretations.

Beyond such simplifications, several behavioral indicators seem to contradict each other across studies. Hanson et al. [4] associated more frequent nose licking with a pleasant taste experience; both Savolainen et al. [39] and van den Bos et al. [21] associated it with a response to a negative or less palatable taste experience; and Bennett et al. [43] interpreted it as a fear response in cats—three fundamentally different interpretations of the same behavior. Sniffing of food shows a similar pattern. Van den Bos et al. [21] and Becques et al. [22] interpreted increased sniffing as hesitation to consume a less palatable diet; Owens et al. [44] found no effect of diet on total sniffing time, a negative correlation between sniffing during the acceptance test and the subsequent preference-to-intake ratio, but no correlation between sniffing during the preference test and the intake ratio; and Kim et al. [46] noted a significant difference in the first sniffing bout between two diets with equal palatability. Latency to eat also showed inconsistent interpretations. Van den Bos et al. [21] and Owens et al. [44] found a rapid approach to food to be indicative of high palatability, whereas Becquest et al. [22] only noted a significant difference during the cats’ final approach to the bowl, when there was no longer a significant difference in intake. Accordingly, Table 1 expands on previous summaries by distinguishing behaviors that are consistently associated with palatability from those with conflicting or context-dependent interpretations.

Several factors contribute to these contradictory findings. Behavioral similarity makes accurate coding of facial-expression detection difficult. Becques et al. [22] and Owens et al. [44] both explicitly acknowledged the difficulty in distinguishing between nose licking and lip licking. However, these two behaviors appear to have opposite palatability interpretations, with lip licking seen as a positive indicator [4,21,39], while nose licking is more generally accepted to be a negative indicator [21,39,43]. If observers cannot reliably distinguish between them, the categorization is compromised. Several studies use fundamentally different test formats (sequential single-bowl tests [21], two-bowl preference tests [4], and five-bowl preference tests [46]), as well as different exposure durations (5–30 min [4,21,44,46], 20 h [22], or until eaten [39]), and subsequently conclude different interpretations of the same behavior. Owens et al. [44] observed that some cats consistently sniff the bowl on the right side more frequently and for longer durations than the bowl on the left, suggesting that side preference may influence sniffing behavior. This problem is compounded in multi-bowl designs [4,44,46], where a behavior such as sniffing could correspond to any specific diet. This methodological fragmentation is also seen in the manner in which behaviors are evaluated, such as “licking/sniffing food” [21], “sniffing/investigating” [47], “length of sniffing” [22], “bouts of sniffing” [44], and “first sniffing bout” [46]. Thus, the contradiction is not just about interpretation; the studies measure different indicators (or combinations of indicators) under different conditions. Similarly, eating speed also shows contradictory interpretations. Owens et al. [44] objectively quantified speed of consumption (g/min) based on food intake and total eating time, whereas Knight and Satchell [47] relied on an owner-completed survey in which consumption speed was not standardized or objectively measured, leaving the results vulnerable to interpretation bias. Individual variability further complicates interpretation and can impact eating patterns [48], sniffing behavior [44], and the hunger response [21]. Consequently, studies with smaller cohorts [44] are more susceptible to individual effects than those with larger cohorts [21,22,39,46]. Beyond design limitations, several gaps exist in the current body of literature. No study has directly compared the impact of sex on behavioral indicators of food palatability, despite evidence that sex-specific behavioral patterns exist in other contexts, such as the repetitive sniffing behavior associated with personality in male cats, but not females [49], and sex-related differences in food preference under specific environmental conditions, such as climate season [50]. These findings suggest that sex may influence how the sensory properties of food are perceived or how palatability is behaviorally expressed. Female cats may, for example, be more sensitive to volatile cues [50]; however, this hypothesis requires further investigation. Future studies should, therefore, test males and females separately and examine sex-by-palatability interactions across multiple behavioral indicators. Another gap is the impact of different diet types, such as raw, wet, or dry foods [10,42]. Physical characteristics of the diet may influence how behavioral indicators of palatability are expressed. For example, lip licking has been associated with greater palatability, predominantly in studies involving wet or other moist foods [4,21,39]; however, it remains unclear whether this response reflects hedonic evaluation alone or may also be influenced by physical characteristics such as moisture and food adherence around the mouth. Future studies should, therefore, directly compare behavioral responses to wet and dry diets while controlling for differences in palatability. Similarly, most studies compare diets that are expected to differ in palatability, yet the literature has not addressed whether behavioral responses become more pronounced as the difference in palatability increases. It should be noted that no behavioral indicator or facial expression is exclusively positive or negative, and that frequency and duration play an important role [24,37]. For example, flattening of the ears is considered a negative reaction and is observed in 89% of negative conditions, but it is also observed in 55% of positive conditions [37]. Similarly, some behavioral indicators, such as sniffing, occur regardless of the presence of food [51]. This means that simply recording the presence or absence of a behavior [39] is insufficient; quantitative measures of frequency and duration are essential, yet some studies [22] only report one or the other, and no studies have established threshold values.

These contradictions have a direct consequence: as the literature is presented currently, behavioral indicators cannot be interpreted as standalone measures of palatability. However, some papers have used these behavioral indicators to determine food palatability from survey data. For example, Knight and Satchell [47] used the existing literature [4,21,22,39] to classify “rapid approach to food” and “eating quickly” as positive indicators of palatability, and “sniffing/investigating food” and “dropping food” as negative indicators. Based on pet owners’ reports of these behaviors, they concluded that vegan and meat-based diets were similarly palatable. However, the literature used to support these classifications is not consistent: Van den Bos et al. [21] and Becques et al. [22] report conflicting findings regarding a rapid approach to food; Hanson et al. [4] and Becques et al. [22] report opposing conclusions regarding sniffing; and Savolainen et al. [39] noted that food dropping cannot be considered an independent indicator because it can only occur when the cat has eaten or is eating, meaning that its higher frequency with palatable food is a consequence of greater consumption rather than an independent measure of palatability. The same behavioral indicators may be positive, negative, or have no meaningful response, depending on the study context. Thus, behavioral data should be interpreted alongside consumption data. For example, a cat licking its lips after eating a large portion of food may indicate good palatability, whereas a cat licking its lips after food refusal is not. Furthermore, lip licking may also simply be the result of cleaning food residue after consuming a particularly messy diet. Without the context, the behavior alone is ambiguous. Just as a sensory lexicon provides a standardized vocabulary for describing the sensory properties of pet food [52,53], this field would also benefit from a standardized ethogram for feline feeding behavior. It should define which behaviors to code, conditions under which they are informative, the quantitative thresholds for frequency and duration, and the food formats for which each indicator has been validated. Standardizing these criteria would improve consistency between studies, reduce contradictory interpretations, and provide a more robust foundation for validating behavioral indicators of feline food palatability.

## 5. Methodological Fragmentation

Many contradictions in the literature cannot be resolved by re-examining individual studies, as the root cause is methodological mismatch. While the existing literature already provides a clear description of the available palatability testing methods [2,9,10,28], several studies [25,36] still apply an evaluation method that does not align with their stated objective. One such methodological mismatch is using the acceptance and preference tests interchangeably. For example, Ilias et al. [36] aimed to determine the “palatability and acceptability” of prescribed diets to “ensure that DSH cats continuously eat”. This is fundamentally an acceptance question, yet a two-bowl preference test was used to compare the therapeutic diets [36]. In another case, Bernachon et al. [25] investigated the “comparative palatability of five supplements”, implying an objective of preference or comparative ranking; however, each cat received only its allocated product, meaning the study measured acceptance within separate groups rather than a direct preference between products [25]. The preference test’s strength lies in discriminating between two products with subtle differences, not in determining whether a single product meets minimum intake thresholds, which is better addressed through acceptance testing [10,54]. This distinction is supported by Hours et al. [55], who reported that voluntary food intake measurements and two-bowl preference test results can yield divergent conclusions about the same diets. Given that these two testing methods address fundamentally different questions, it is surprising that only a few published studies have performed both tests in sequence for a more comprehensive assessment [24,55,56]. Similarly, the liking test [57], a theoretically ideal position between acceptance and preference testing, has also not been adopted by the research community and is only mentioned in review papers [10,37]. Unlike the Concurrent Schedule Paradigm [58,59] and the Concurrent Paired-Associative Preference [60,61], whose limited adoption can be attributed to practical constraints, the liking test’s absence from the literature is harder to explain. It requires no specialized equipment, uses a standard one-bowl format, and was specifically designed to be feasible with expert panels [57]. Its absence represents an independent validation gap.

**Figure 3:**
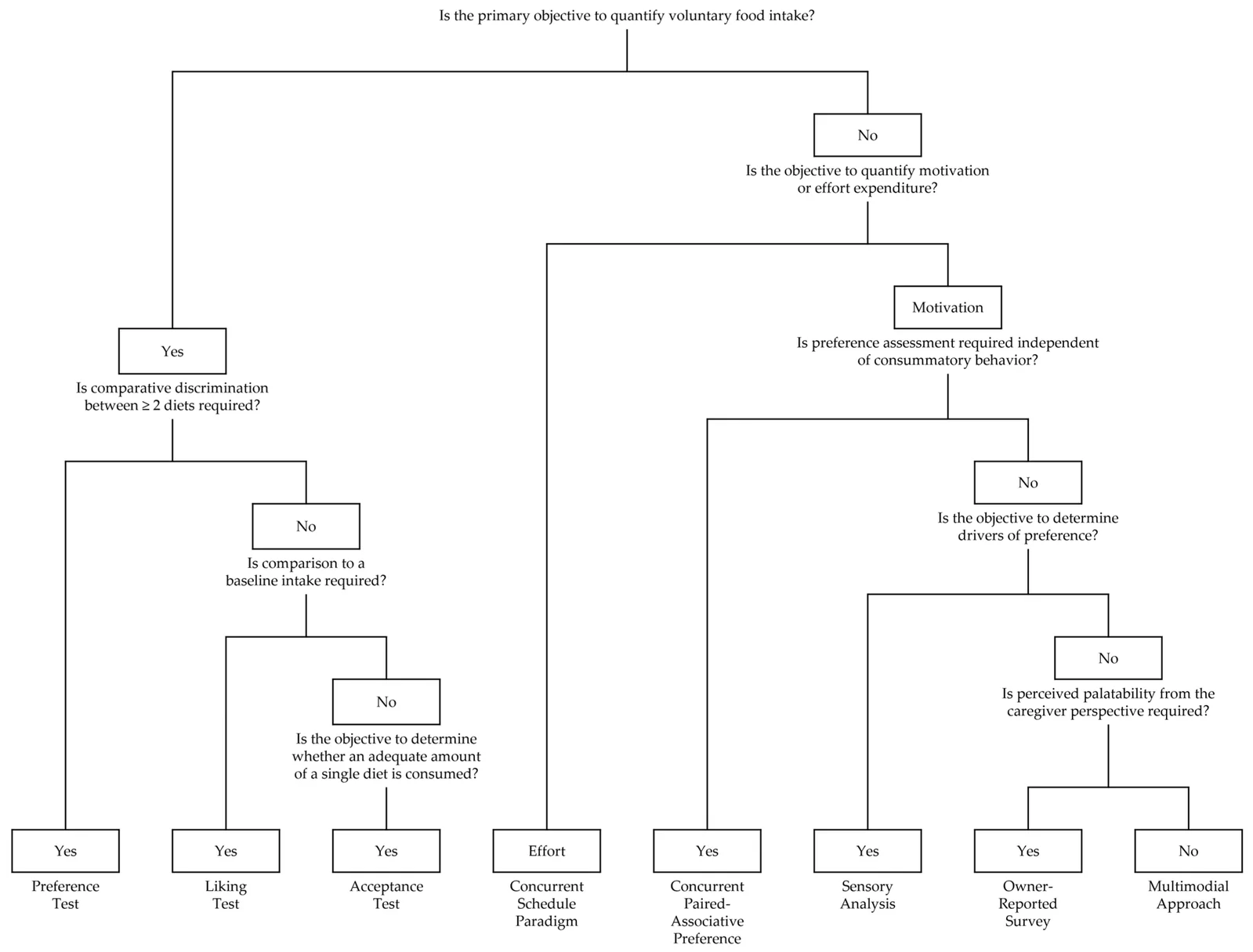
Decision framework for palatability assessment methodologies under resource constraints.

Part of the methodological fragmentation in the literature is driven by resource constraints. Pet food producers and academic laboratories may lack the time, animals, infrastructure, and/or funding to run multiple test formats to ensure a complete palatability picture [28]. Recognizing this reality, Figure 3 proposes a decision framework to guide method selection based on the primary research objective, with the branching questions representing the research questions applicable to each methodology. This enables researchers to identify the single most informative test for their specific question, rather than defaulting to convention.

Another methodological factor contributing to contradictory palatability findings is the choice of sample size and test duration. According to Tobie et al. [10], a preference test on an expert panel requires at least 30 cats to ensure statistical robustness, and in-home testing requires 100. However, more recent work indicates that the ideal sample size depends on the statistical framework and test methodology, as factors such as cat and food characteristics, degree of adaptation or training, and testing environment may influence variability and, consequently, the number of animals required [62,63]. Pires et al. [62] used power analysis to demonstrate that a preference test with 23 cats is sufficient to detect a difference of 0.10 in the intake ratio with 75% power, whereas Bos et al. [63] used a precision-based approach to justify a sample size of 17–23 to reach a 10% margin of error (MoE) in two-bowl preference tests, depending on the contrast between the dry extruded foods tested. For acceptance tests, Bos et al. [63] noted greater variability, with 12–43 cats required to achieve the same MoE. These are two fundamentally different approaches. Statistical power is the probability of rejecting a false null hypothesis [64]; thus, power analysis can be used to determine whether a study is adequately powered to detect a specified effect, whereas MoE is used to determine how precisely the mean intake can be estimated [65]. Currently, no study has provided a statistically derived sample size for one-bowl acceptance tests on expert panels. Bos et al. [63] noted that “a shorter study length with a greater number of cats is superior”, as increasing the test duration may result in dropout, reducing accuracy. Both Pires et al. [62] and Bos et al. [63] indicate that a single test day may be sufficient; however, this should be distinguished from the need for pre-test adaptation or familiarization, which may be required when animals are not accustomed to the testing procedure. Despite this, many studies [36,66,67,68] continue to use extended protocols—5–10 days with panels of 10 or fewer. Such designs may provide limited ability to accommodate inter-individual variability, while extended testing may additionally increase the risk of dropout-related bias. In contrast, industry practice and several academic studies [40,69,70,71] typically use a panel of 20 cats tested over 2 days, resembling the recommendation of Bos et al. [63] more closely. The importance of reporting sample size rationale and statistical framework alongside the study results is often overlooked. This was highlighted by Pires et al. [62], who found that, of the 16 studies included in their systematic review, only three reported sufficient information to allow a full assessment of the statistical power of cat food preference tests. This lack of transparency likely contributes to the contradictions between studies, and it is therefore recommended that an appropriate sample size be determined by using power analysis based on the expected difference in intake ratio, anticipated variability, significance level, and desired statistical power [62], rather than adopting a fixed number of cats.

Beyond sample size and test duration, a deeper form of methodological fragmentation may arise through the propagation of claims across secondary sources without verification against the original evidence. Seasonal variation in feline food intake provides a clear example. Watson et al. [9] stated that cats are prone to “eating less in the winter than in the summer”, citing Tobie et al. [10], who similarly stated that cats eat “less during winter”. However, Tobie et al. [10] cited Serisier et al. [27] as the underlying primary evidence, whereas Serisier et al. [27] actually reported the opposite pattern, with food intake increasing during winter. This direction is also consistent with the findings of Alegría-Morán et al. [50], who reported increased food intake under colder conditions. Thus, the issue illustrated here is not simply disagreement among studies, but the propagation of an incorrectly reported direction of effect from a secondary source into the subsequent literature. Another example includes the statement of Watson et al. [9] that “cats are driven to foods with a high protein and fat content and avoid carbohydrate-rich foods” by citing Hewson-Hughes [72] and Salaun et al. [73] without acknowledging that the high-carbohydrate target identified by Hall et al. [74] was specifically observed under conditions of equal palatability. This contributes to methodological fragmentation by creating an inconsistent evidence base, making it difficult to compare studies, accurately interpret findings, and develop robust experimental designs.

## 6. Owner Perception Paradox

The central contradiction of feline palatability is that the pet owner who chooses and purchases the pet food is not the final consumer. Pet food is seen as an easy opportunity to create a positive experience in the everyday life of a pet [4]. Pet owners interpret their pet’s willingness to eat as an immediate indicator of satisfaction [75], and when pets reject their food, owners feel they are not providing adequate care and consequently seek a better alternative [2,76]. However, what happens when the pet does not outright refuse the food but also does not find it maximally palatable? The cat adjusts its intake downward within an acceptable range, and the owner assumes that palatability is adequate, unaware that the cat would consume significantly more of a preferred alternative. This is what we term the “owner perception paradox”; the sensory preferences, cognitive biases, and emotional attachments of the owner override the biological reality of the cat’s preferences.

Owners and cats perceive food through different sensory channels. Often, pet owners evaluate the food based on appearance and aroma only [36,77,78], while cats evaluate the complete palatability experience, including aroma, taste, and texture [38,39,79,80]. The physiological differences are substantial. Cats exhibit 21 cm^2^ of olfactory epithelia, compared to the 3–4 cm^2^ in humans [36], and their sense of smell is roughly fourteen times more sensitive [81], with oxidation detection alone being a hundred times more acute than in humans [82]. The vomeronasal organ further allows them to detect volatile compounds that are imperceptible to human noses [82,83]. Cats possess ≈ 470 taste buds, considerably fewer than humans (≈10,000), suggesting lower taste sensitivity [3]. However, this impression is misleading, as cats have a heightened sensitivity for certain bitter compounds [4,29,37], their umami receptors are not activated by the same amino acids [84], and they lack the functional receptor for sweet taste entirely [18,85], meaning that the sugary aromas that often attract human attention in pet food marketing are invisible to the feline palate. Thus, the sensory attributes used by the owner to select a diet for their cat are fundamentally different from the sensory attributes used by the cat to consume the food.

Ilias et al. [36] demonstrated this divergence by comparing the odor scores of a human panel to the consumption outcome of a cat panel, across four prescribed diets. Both species agreed that the hypersensitivity diet was least preferred, but their discriminatory capacity differed thereafter. Cats produced a clear rank ordering across the remaining three diets based on intake (although this was not statistically significant), whereas the human odor panel rated the three remaining diets as broadly equivalent [36]. Similarly, owner surveys have been a widely used approach to assess pet food palatability from the owner’s perspective since at least the early 1990s [86,87,88,89,90], but they also provide limited interpretation due to bias [9,47], inconsistent judgment [86], and variable pet behavior interpretations [91]. This is evident in the study conducted by Sanderson et al. [86], where owners were unknowingly given the same diet to feed their dog in consecutive periods, and 27% gave inconsistent responses. While this study involved dogs rather than cats, the underlying principle still applies. Similarly, Bos et al. [63] found that the owners’ categorization of their cat’s pickiness did not correspond with their food intake. Cats categorized as “easy” eaters by their owners had an overall lower food intake compared to “difficult” or “very difficult” eaters. Yet, instead of owners being uncertain about their cat’s preferences, they actively project their own preferences onto their cats. Di Donfrancesco et al. [92] found a correlation of 0.93 between how much owners liked a product and how much they predicted their pet would like it. However, the drivers of owner liking (appearance, color, and aroma) are not the drivers of pet intake (aroma, taste, and texture). This projection is reinforced by the growing influence of pet humanization [93,94,95]. In a survey of 1380 cat owners, Mace et al. [96] noted that the most important feature influencing willingness to purchase alternative diets was good health outcomes, limited to the owner’s knowledge level. Thus, their purchases reflect their own values (sustainability, organic ingredients, or human-grade quality) rather than the cat’s nutritional and sensory requirements [93,96,97]. This leads to specific, demonstrable misalignments. Owners frequently prefer diets marketed as natural or free from additives [96,98], unaware that removing certain ingredients, such as sugars, compromises Maillard-driven flavor generation [99,100]. Similarly, owners prefer diets with higher protein and fresh meat content [75], unaware that the gravy portion of a chunks-and-gravy diet drives palatability [101]. A survey by Erzurum and Kayar [75] captures this contradiction: pet owners rated “pet preference (palatability)” as their highest selection criteria, and “visual appearance” as the lowest. Owners believe they are choosing based on their cat’s preference. Yet, as Di Donfrancesco et al. [92] demonstrated, what the owner believes their cat prefers is indistinguishable from what the owner personally prefers. The consequences are also seen in the academic literature. Knight and Satchell [47] surveyed 1135 cat owners and found that diet (conventional, raw, and vegan) made little difference to food-oriented behavior, concluding that vegan pet foods are as palatable as conventional meat or raw meat diets. This finding was characterized by subsequent reviewers as “extremely subjective” [9] and contributes to “ambiguous results” [11]. Owner reporting does not merely introduce noise; it can actively flatten real palatability differences. Importantly, the owner perception paradox is unlikely to be uniform across owners with different feeding experiences or cultural backgrounds. Owners with greater knowledge may weigh product attributes differently [97], while caregivers who actively monitor food intake may be better positioned to recognize acceptance and preference [24]. Cultural differences further shape responses to pet food odors [78] and the importance assigned to attributes such as naturalness, freshness, sustainability, palatability, brand, and veterinary advice [28,75,96]. However, demographic effects are not consistent across populations; for example, age and education were unrelated to purchasing behavior in one Turkish sample [75], whereas older guardians were less influenced by price and convenience in a predominantly UK and European survey [96]. Thus, owner perception should not be treated as a single, stable source of bias. An important research gap is to determine which owner characteristics increase or reduce alignment between perceived and objectively measured feline palatability across different cultural and feeding contexts.

The resolution to this paradox is not to exclude owner interpretation entirely, as owners remain the ultimate decision-makers. Rather, it is to recognize that owner evaluation and feline consumption measure fundamentally different constructs. Owner surveys capture purchasing intent and owner satisfaction; consumption testing captures the cat’s hedonic response. These are complementary measures, not interchangeable. Owner perception should not be assumed to reflect pet palatability, nor should pet palatability be assumed to result in commercial success. Successful pet food products depend on satisfying both audiences, whose sensory channels, cognitive frameworks, and evaluative criteria are misaligned.

## 7. Limitations

This review has several limitations. First, a narrative review involves synthesizing evidence across different studies, meaning that the identification and grouping of themes involve some degree of subjective interpretation by the authors. Second, the primary search was limited to the Scopus database and English-language publications, although additional studies were identified through targeted searches and citation tracking. Finally, the review incorporates a heterogeneous body of evidence, including experimental, methodological, and review publications, which may limit direct comparison between studies.

## 8. Conclusions and Future Research Directions

Despite considerable advances over recent decades, feline food palatability research continues to be hindered by methodological and conceptual inconsistencies. This critical narrative review demonstrates that variable conceptual definitions, experimental execution, and interpretation of behavioral indicators limit the development of a reliable framework for evaluating feline food satisfaction.

Thus, future research should shift from a descriptive to a prescriptive approach, providing methodological and interpretation guidance instead of only identifying or summarizing findings. The decision tree proposed in this review may help align trial designs with their specific research objectives, while routine reporting of statistical power and/or precision would improve the robustness and reproducibility of palatability claims. Similarly, separating owner impressions from feline responses and adopting a standardized ethogram for behavioral indicators will resolve ambiguity and improve comparability between studies.

Future research priorities can be structured into three areas. Basic research should focus on evaluating test–retest reliability, assessing the influence of food format (e.g., dry, wet, and semi-moist) and animal characteristics (e.g., age, sex, neuter status, and health status), and comparing owner perceptions with objectively measured feline responses. Methodological standardization should prioritize validating a standardized feline feeding ethogram, assessing whether the proposed decision framework improves the selection of palatability methodologies for different research objectives, and developing minimum reporting recommendations for feline palatability studies. Finally, industrial application should evaluate the applicability of these approaches across different products, testing facilities, and commercial settings. By harmonizing how findings are reported and interpreted, the industry can finally transform isolated observations into a coherent and actionable evidence base.

## Figures and Tables

**Table 1:** Summary of positive, negative, and inconsistent behavioral indicators associated with feline food palatability across studies.

Behavioral Indicator	No. of Studies	Overall Interpretation	Study Interpretation	Behavioral Metric	Food Type	Experimental Context	Reference
Latency to eating	3	Inconsistent	Positive	Decreased latency before consumption	Wet foods	One-bowl acceptance 18 cats (Groups B & C), 1 × 10 min exposure	[21]
			Positive	Decreased latency before consumption; negatively correlated with intake ratio	Raw chicken	One-bowl acceptance 7 cats, 20 × 30 min exposures Two-bowl preference 6 cats, 2 × 30 min exposures	[44]
			No correlation	Duration before consumption	Dry foods	One-bowl acceptance 17 cats, 2 × 20 h exposures	[22]
Speed of consumption	2	Inconsistent	Negative	Increased speed during consumption	Raw chicken	One-bowl acceptance 7 cats, 20 × 30 min exposures	[44]
			No correlation	Speed during consumption	Dry foods	One-bowl acceptance 17 cats, 2 × 20 h exposures	[22]
Flicking ears backwards	1	Negative	Negative	Increased occurrence before consumption	Commercial cat foods and various human foods	One-bowl acceptance 34 cats, 6 × until eaten/refused	[39]
Ears upwards/forwards	1	Positive	Positive	Increased duration during sampling and consumption	Aqueous tastant solutions	Two-bowl preference 13 cats, 2 × 5 min exposures	[4]
Ears lowered/outwards	1	Inconsistent	Inconsistent	Increased duration during sampling	Aqueous tastant solutions	Two-bowl preference 13 cats, 2 × 5 min exposures	[4]
Mouth smacking	1	Positive	Positive	Increased frequency during sampling	Aqueous tastant solutions	Two-bowl preference 13 cats, 2 × 5 min exposures	[4]
Tongue protrusions	1	Positive	Positive	Increased frequency during sampling	Aqueous tastant solutions	Two-bowl preference 13 cats, 2 × 5 min exposures	[4]
Tongue-protrusion gapes	1	Negative	Negative	Increased frequency overall	Aqueous tastant solutions	Two-bowl preference 13 cats, 2 × 5 min exposures	[4]
Lip licking	3	Positive	Positive	Increased frequency after consumption	Wet foods	One-bowl acceptance 15 cats (Groups A & C), 1 × 10 min exposure	[21]
			Positive	Increased occurrence overall	Commercial cat foods and various human foods	One-bowl acceptance 34 cats, 6 × until eaten/refused	[39]
			Positive	Increased frequency overall	Aqueous tastant solutions	Two-bowl preference 13 cats, 2 × 5 min exposures	[4]
Nose licking	3	Inconsistent	Negative	Increased occurrence before consumption	Commercial cat foods and various human foods	One-bowl acceptance 34 cats, 6 × until eaten/refused	[39]
			Positive	Increased frequency during sampling	Aqueous tastant solutions	Two-bowl preference 13 cats, 2 × 5 min exposures	[4]
			Negative	Increased duration and frequency after consumption	Wet foods	One-bowl acceptance 15 cats (Groups A & C), 1 × 10 min exposure	[21]
Face licking	1	No correlation	No correlation	Frequency overall	Dry foods	One-bowl acceptance 17 cats, 2 × 20 h exposures	[22]
Paw licking	2	No correlation	No correlation	Frequency and correlation with intake ratio	Raw chicken	One-bowl acceptance 7 cats, 20 × 30 min exposures Two-bowl preference 6 cats, 2 × 30 min exposures	[44]
			No correlation	Duration and frequency after consumption	Wet foods	One-bowl acceptance 23 cats (Groups A, B, & C), 1 × 10 min exposure	[21]
Empty-bowl licking	1	No correlation	No correlation	Duration after consumption	Raw chicken	One-bowl acceptance 7 cats, 20 × 30 min exposures	[44]
Licking/sniffing Food	1	Negative	Negative	Increased duration before consumption	Wet foods	One-bowl acceptance 15 cats (Groups A & C), 1 × 10 min exposure	[21]
Licking/sniffing empty bowl	2	Inconsistent	Positive	Increased duration after consumption	Wet foods	One-bowl acceptance 23 cats (Groups A, B, & C), 1 × 10 min exposure	[21]
			No correlation	Frequency after consumption does not correlate with intake ratio	Raw chicken	One-bowl acceptance 7 cats, 20 × 30 min exposures Two-bowl preference 6 cats, 2 × 30 min exposures	[44]
Sniffing food	4	Inconsistent	Inconsistent	Duration, frequency, and correlation with intake ratio	Raw chicken	One-bowl acceptance 7 cats, 20 × 30 min exposures Two-bowl preference 6 cats, 2 × 30 min exposures	[44]
			Positive	Increased duration during sampling	Aqueous tastant solutions	Two-bowl preference 13 cats, 2 × 5 min exposures	[4]
			Negative	Increased duration during first visit	Dry foods	One-bowl acceptance 17 cats, 2 × 20 h exposures	[22]
			Inconsistent	Increased frequency of first sniffing bout	Dry foods	Five-bowl preference 16 cats, 1 × 5 min exposure	[46]
Sniffing bowl	1	Positive	Positive	Increased occurrence overall	Commercial cat foods and various human foods	One-bowl acceptance 34 cats, 6 × until eaten/refused	[39]
Sniffing table/floor	3	Inconsistent	No correlation	Occurrence overall	Commercial cat foods and various human foods	One-bowl acceptance 34 cats, 6 × until eaten/refused	[39]
			No correlation	Duration, frequency, and correlation with intake ratio	Raw chicken	One-bowl acceptance 7 cats, 20 × 30 min exposures Two-bowl preference 6 cats, 2 × 30 min exposures	[44]
			Inconsistent	Increased duration after consumption	Wet foods	One-bowl acceptance 8 cats (Group B), 1 × 10 min exposure	[21]
Eating in a sitting position	1	No correlation	No correlation	Duration during consumption	Dry foods	One-bowl acceptance 17 cats, 2 × 20 h exposures	[22]
Standing on all four paws	1	No correlation	No correlation	Duration and frequency during consumption	Wet foods	One-bowl acceptance 23 cats (Groups A, B, & C), 1 × 10 min exposure	[21]
Sitting upright	1	No correlation	No correlation	Duration and frequency during consumption	Wet foods	One-bowl acceptance 23 cats (Groups A, B, & C), 1 × 10 min exposure	[21]
Half-open eyes	1	Positive	Positive	Increased duration during sampling and consumption	Aqueous tastant solutions	Two-bowl preference 13 cats, 2 × 5 min exposures	[4]
Pawing floor/head shaking/limb flailing	1	Inconsistent	Inconsistent	Frequency and correlation with intake ratio	Raw chicken	One-bowl acceptance 7 cats, 20 × 30 min exposures Two-bowl preference 6 cats, 2 × 30 min exposures	[44]
Flicking of tail	1	Negative	Negative	Increased occurrence overall	Commercial cat foods and various human foods	One-bowl acceptance 34 cats, 6 × until eaten/refused	[39]
Shaking paw	1	No correlation	No correlation	Occurrence overall	Commercial cat foods and various human foods	One-bowl acceptance 34 cats, 6 × until eaten/refused	[39]
Twitching back skin	1	No correlation	No correlation	Occurrence overall	Commercial cat foods and various human foods	One-bowl acceptance 34 cats, 6 × until eaten/refused	[39]
Face grooming	2	Inconsistent	No correlation	Frequency and correlation with intake ratio	Raw chicken	One-bowl acceptance 7 cats, 20 × 30 min exposures Two-bowl preference 6 cats, 2 × 30 min exposures	[44]
			Positive	Increased duration and frequency after consumption	Wet foods	One-bowl acceptance 15 cats (Groups A & C), 1 × 10 min exposure	[21]
Body grooming	3	Inconsistent	Negative	Increased occurrence overall	Commercial cat foods and various human foods	One-bowl acceptance 34 cats, 6 × until eaten/refused	[39]
			No correlation	Duration and frequency after consumption	Wet foods	One-bowl acceptance 23 cats (Groups A, B, & C), 1 × 10 min exposure	[21]
			No correlation	Frequency and correlation with intake ratio	Raw chicken	One-bowl acceptance 7 cats, 20 × 30 min exposures Two-bowl preference 6 cats, 2 × 30 min exposures	[44]

## Data Availability

No new data were created or analyzed in this study. Data sharing is not applicable to this article.
